# Decoding the Hexosamine Biosynthesis Pathway: Implications for Novel Therapeutic Strategies in Sarcoma

**DOI:** 10.1002/jcp.70182

**Published:** 2026-05-11

**Authors:** Pegah Rahimizadeh, Richard Miallot, Chelsea De Bellis, Philippe Jolivet, Joanna Przybyl

**Affiliations:** ^1^ Division of Surgical and Interventional Sciences McGill University Montreal Canada; ^2^ Cancer Research Program The Research Institute of the McGill University Health Centre Montreal Canada; ^3^ Department of Surgery McGill University Montreal Canada

**Keywords:** glycosylation, hexosamine biosynthesis pathway, O‐GlcNAcylation, sarcoma

## Abstract

Cancer cells rewire their metabolism to sustain a high proliferation rate. Sensing external cues is essential to match the metabolic fluxes of the cells to the external stimuli. As part of the glucose metabolism, the hexosamine biosynthesis pathway (HBP) is considered a nutrient‐sensing pathway. The HBP produces UDP‐GlcNAc, a key precursor for N‐linked glycosylation, O‐linked glycosylation, and O‐GlcNAcylation. These post‐translational modifications can influence protein folding, interactions, and subcellular localization. Altered glycosylation of oncogenic proteins has been linked to the acquisition of malignant properties. In this review, we outline the current knowledge of molecular alterations and the prognostic role of the expression of HBP enzymes in sarcoma. We catalog the known sites of N‐/O‐linked glycosylation and O‐GlcNAc modifications in molecular drivers of mesenchymal tumors, and discuss the potential effect of these modifications on protein function. We also summarize the studies that examined the effect of the HBP inhibitors in preclinical models of cancer, and explore the potential of the HBP inhibition as a novel therapeutic approach for sarcoma. Finally, we present recent progress in drug development for targeting the HBP, and delineate the key technological innovations needed to accelerate the preclinical and clinical research on pharmacological inhibition of the HBP.

**Abbreviations**
2‐DG: 2‐deoxyglucose
5‐FU: 5‐fluorouracil
AA: amino acids
Acetyl‐CoA: acetyl coenzyme A
Ac‐5 s‐GlcNAc: acetylated 5S‐GlcNAc
AML: acute myeloid leukemia
AOM: azoxymethane
Asn: asparagine
AT/RT: atypical teratoid/rhabdoid tumors
Azaserine: O‐diazoacetyl‐L‐serine
BADGP: benzyl 2‐acetamido‐2‐deoxy‐α‐D‐galactopyranoside
CDK: cyclin‐dependent kinases
CGC: cancer gene census
CoA: coenzyme A
COSMIC: Catalogue of Somatic Mutations in Cancer
DLBCL: diffuse large B‐cell lymphoma
DON: 6‐diazo‐5‐oxo‐L‐norleucine
DSS: dextran sulfate sodium
ECM: extracellular matrix
ER: endoplasmic reticulum
F6P: fructose‐6‐phosphate
G6P: glucose‐6‐phosphate
GALE: UDP‐glucose 4‐epimerase
GalNAc: N‐acetylgalactosamine
GFPT1/GFAT1: glutamine‐‐fructose‐6‐phosphate aminotransferase [isomerizing] 1
GFPT2/GFAT2: glutamine‐‐fructose‐6‐phosphate aminotransferase [isomerizing] 2
GlcNAc: N‐acetylglucosamine
GlcNAc‐1P: N‐acetylglucosamine‐1‐phosphate
GlcNAc‐6P: N‐acetylglucosamine‐6‐phosphate
GlcN‐6P: glucosamine‐6‐phosphate
GLUT: glucose transporters
GNA1/GNPNAT1: glucosamine‐6‐phosphate N‐acetyltransferase 1
HBP: hexosamine biosynthesis pathway
HTRF: homogeneous time‐resolved fluorescence
Ig: immunoglobulin
IGF‐1R: insulin‐like growth factor 1 receptor
iPTMnet: integrated post‐translational modification network
KDR: kinase insert domain receptor
MEK: mitogen‐activated protein kinase kinase
MICA: major histocompatibility class I‐related chain molecule A
OGA: O‐GlcNAc hydrolase, O‐GlcNAcase
OGT: O‐GlcNAc transferase
O‐GlcNAc: O‐linked β‐N‐acetylglucosamine
O‐GlcNAcylation: O‐linked‐N‐acetylglucosaminylation
PD1: programmed cell death protein 1
PDAC: pancreatic ductal adenocarcinoma
PD‐L1: programmed death‐ligand 1
PGM3: phosphoacetylglucosamine mutase 3
PPi: inorganic pyrophosphate
PugNAc: 1,5‐hydroximolactone
ROCK2: Rho‐associated coiled‐coil forming protein kinase
ROS: reactive oxygen species
Ser: serine
SMART: simple modular architecture research tool
TCGA: the cancer genome atlas
Thr: threonine
TK: thioketal
TRAIL: TNF‐related apoptosis‐inducing ligand
UAP1: UDP‐N‐acetylglucosamine pyrophosphorylase 1
UDP: uridine diphosphate
UDP‐GalNac: uridine diphosphate N‐acetylgalactosamine
UDP‐GlcNAc: uridine diphosphate N‐acetylglucosamine
UTP: uridine 5′‐triphosphate

## 1 Background

Cancer cells maintain enhanced proliferation rate in a nutrient‐deprived environment through acquired alterations in growth signaling, resistance to cell death, and cell cycle regulation (Comito et al. 2020; Garcia‐Bermudez et al. 2020). Cells need to efficiently use the sources of carbon, fatty acids, and sugar to balance cell proliferation with hypoxia, changes in the redox status, and resistance to restricted access to nutrients (Oronsky et al. 2014; Yin et al. 2012). It has been proposed that targeting the ability of cancer cells to sense their surrounding environment may provide therapeutic benefits (Hiscox et al. 2011; Sounni and Noel 2013). Cancer cell metabolism often relies on high glucose consumption and limited mitochondrial activity (Hay 2016). Scarcity of substrates in the tumor microenvironment may cause changes in the functioning of cell signaling pathways. To overcome these challenges, cancer cells undergo metabolic rewiring to maintain cellular plasticity. For instance, in low‐glucose conditions, cancer cells can uptake more glutamine, lactate, and fatty acids as alternative sources of carbon (Corbet and Feron 2017; Zheng 2012).

Soft tissue and bone sarcomas are tumors of mesenchymal origin that encompass over 70 different histological subtypes that are associated with distinct clinical behavior and require different therapeutic strategies (WHO 2020). Surgery remains the treatment of choice for most sarcomas (WHO 2020). Chemotherapy and radiation therapy are considered in addition to surgery in patients with high risk for local recurrence and distant metastasis (WHO 2020; Grünewald et al. 2020). The response to standard chemo‐ and radiotherapy is variable, and only a few histological subtypes of sarcoma show response to targeted therapy and immunotherapies (WHO 2020; Grünewald et al. 2020; Wood et al. 2024; Schöffski et al. 2014). Thus, substantial efforts are focused on the development of new combinations of therapeutic agents to increase sensitivity to standard therapy and improve treatment efficacy (Schöffski et al. 2014). Sarcomas show highly active metabolic phenotypes and elevated levels of transcriptional signatures associated with glucose consumption and glycolysis compared to other cancer types (Shaw et al. 1988; Miallot et al. 2021). Thus, modulating metabolic pathways in combination with other therapies could offer a promising approach for developing effective therapies for sarcoma (Miallot et al. 2021).

Therapeutic targeting of the hexosamine biosynthesis pathway (HBP) has emerged as a relevant and promising approach in cancer (Lam et al. 2021). Activation of the HBP is frequently observed during cancer development, leading to altered patterns of N‐/O‐linked glycosylation and O‐GlcNAcylation (O‐linked‐N‐acetylglucosaminylation) (Chiaradonna et al. 2018; Akella et al. 2019). The HBP is a therapeutic vulnerability in cancer because inhibition of the HBP enzymes can suppress tumor cell growth, modulate the immune response, and sensitize tumor cells to conventional treatment (Lam et al. 2021). Emerging evidence highlights the role of the HBP also in the etiology of selected histological subtypes of sarcoma. Here, we review the current state of knowledge on the role of the HBP in sarcoma and cancer, based on the peer‐reviewed original research studies and review articles published between 1957 and 2024 and indexed in PubMed. In this review, we also examine the prevalence and potential functional impact of glycosylation in molecular drivers of sarcoma, based on the data from curated, peer‐reviewed, and publicly available databases of post‐translational modifications and functional annotation of proteins. Finally, we outline the currently available strategies for therapeutic targeting of the HBP, and discuss the potential of HBP inhibition as a novel therapeutic approach for sarcoma.

## 2 Hexosamine Biosynthesis Pathway (HBP)

**Figure 1:**
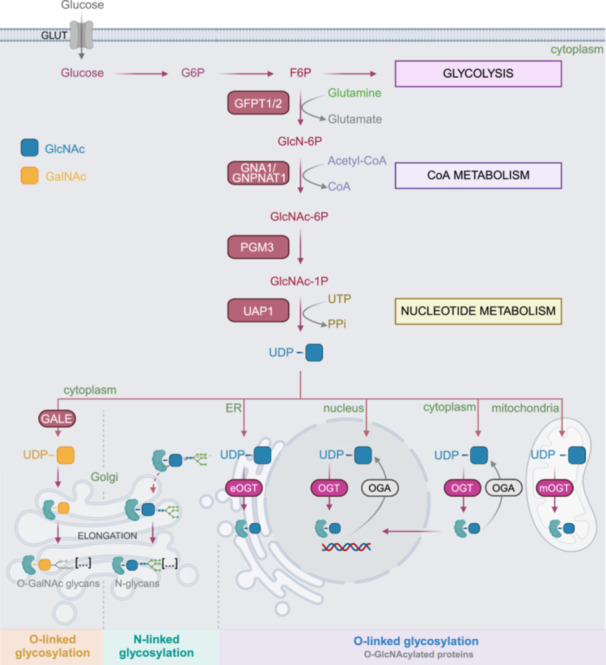
Enzymatic reactions of the HBP and downstream protein modifications. In the HBP, F6P is converted into UDP‐GlcNAc through a series of enzymatic reactions catalyzed by GFPT1/2, GNPNAT1, PGM3, and UAP1. UDP‐GlcNAc is the common precursor for O‐ and N‐linked glycosylation, which takes place in the cytoplasm. UDP‐GlcNAc is also the precursor for O‐GlcNAcylation, which can take place in multiple subcellular compartments, including the cytoplasm, nucleus, endoplasmic reticulum, and mitochondria. (Acetyl‐CoA, acetyl coenzyme A; CoA, coenzyme A; ER, endoplasmic reticulum; F6P, fructose‐6‐phosphate; GALE, UDP‐glucose 4‐epimerase; GFPT, glutamine–D‐fructose‐6‐phosphate transaminase; GlcNAc‐1P, N‐Acetylglucosamine‐1‐phosphate; GlcNAc‐6P, N‐Acetyl‐D‐glucosamine 6‐phosphate; GlcN‐6P, glucosamine 6‐phosphate; GLUT, glucose transporters; G6P, glucose‐6‐phosphate; GNPNAT1, glucosamine 6‐phosphate N‐acetyltransferase 1; OGA, O‐GlcNAcase; OGT, O‐GlcNAc transferase (eOGT ‐ endoplasmic, mOGT ‐ mitochondrial); PGM3, phosphoacetylglucosamine mutase 3; PPi, inorganic pyrophosphate; UAP1, UDP‐N‐acetylhexosamine pyrophosphorylase; UDP‐GalNAc, uridine diphosphate N‐acetylgalactosamine; UDP‐GlcNAc, uridine diphosphate N‐acetylglucosamine; UTP, Uridine 5′‐triphosphate).

The HBP is a metabolic pathway that is active in most cells within the cytoplasm. The first two steps of the HBP and glycolysis are shared by both pathways, in which glucose undergoes conversion to fructose‐6‐phosphate (F6P) (Figure 1). Next, most of F6P is directed to glycolysis, and approximately 2%–5% of F6P enters the HBP, as estimated in non‐cancerous cells (Marshall et al. 1991). Through the HBP, F6P is converted to UDP‐N‐acetylglucosamine (UDP‐GlcNAc) via a series of enzymatic reactions, as reviewed by Paneque et al. (2023) (Figure 1). In the first step, glutamine‐fructose‐6‐phosphate amidotransferase (GFPT – EC 2.6.1.16) catalyzes the isomerization reaction, converting glucose‐6‐phosphate and L‐glutamine into glucosamine‐6‐phosphate (GlcN‐6P) and glutamate. This aminotransferase is encoded by two highly homologous genes, *GFPT1* and *GFPT2*. Following this step, glucosamine‐6‐phosphate N‐acetyltransferase 1 (GNA1/GNPNAT1 – EC 2.3.1.4) facilitates the acetylation process by transforming GlcN‐6P and acetyl coenzyme A into N‐acetylglucosamine‐6‐phosphate (GlcNAc‐6P) and coenzyme A. The third step involves phosphoacetylglucosamine mutase 3 (PGM3 – EC 5.4.2.3), which catalyzes the isomerization of GlcNAc‐6P to N‐acetylglucosamine‐1‐phosphate (GlcNAc‐1P). Lastly, UDP‐N‐acetylglucosamine pyrophosphorylase 1 (UAP1 – EC 2.7.7.23) catalyzes the phosphorylation reaction, which converts GlcNAc‐1P and UTP into UDP‐GlcNAc and pyrophosphate (Figure 1). As such, the HBP is tightly linked to glycolysis, coenzyme A metabolism, and nucleotide metabolism. UDP‐GlcNAc is the precursor for O‐linked glycosylation, after the conversion into UDP‐GalNAc catalyzed by UDP‐glucose 4‐epimerase (GALE – EC 5.1.3.2). UDP‐GlcNAc also serves as the precursor for N‐linked glycosylation, which is catalyzed by glycosyltransferases, and for O‐GlcNAcylation, which is catalyzed by O‐linked N‐acetylglucosaminyltransferase (OGT – EC 2.4.1.255). O‐GlcNAc (O‐Linked β‐N‐acetylglucosamine) modification can be removed by O‐GlcNAc hydrolase (OGA – EC 3.2.1.169) (Figure 1).

## 3 Prognostic Value of Expression of the HBP Components in Sarcoma

Several groups, including ours, have investigated the potential prognostic value of expression of the HBP components in selected histological subtypes of sarcoma. We have previously demonstrated a significant enrichment of RNA expression of multiple genes encoding the HBP enzymes in a subset of leiomyosarcoma (Tolwani et al. 2021). We have also demonstrated that expression of the GFPT2 protein in leiomyosarcoma is associated with poor prognosis and may be associated with increased glucose uptake in these tumors (Tolwani et al. 2021). Su et al. (2022) demonstrated a significant enrichment of expression of the HBP genes in a subset of osteosarcomas, and elevated expression of the HBP genes was associated with poor prognosis. These authors also showed that the UAP1 protein expression was associated with unfavorable overall and metastasis‐free survival in osteosarcoma patients (Su et al. 2022). Another study showed that a decreased expression of the OGA protein is associated with poor response to neoadjuvant chemotherapy and shorter metastasis‐free survival in osteosarcoma patients (Sombutthaweesri et al. 2022). These studies demonstrated that the expression levels of the HBP enzymes may serve as prognostic biomarkers in leiomyosarcoma and osteosarcoma.

## 4 Molecular Mechanisms Regulating HBP Enzymes

The abundance and activity of metabolic enzymes can be regulated through multiple mechanisms, including genomic alterations, transcriptional programs, and post‐translational modifications, often in response to nutrient status and cellular stress.

At the genomic level, several studies reported *UAP1* amplification in well‐differentiated liposarcoma, myxoid liposarcoma, and pleomorphic liposarcoma, and chromosomal rearrangements involving *OGT* and *OGA* in soft tissue tumors (Kanojia et al. 2015; Torrence et al. 2021; Ieremia and Thway 2014; Suster et al. 2020; Carter et al. 2014; Liu, Sukov et al. 2019). Nevertheless, the possible effect of these alterations on the activation of these genes remains unknown. To further explore possible genomic alterations in the genes encoding the key HBP enzymes in sarcoma, we queried the publicly available data in the cBioPortal from The Cancer Genome Atlas (TCGA) generated from 206 adult soft tissue sarcomas representing six different histological subtypes (Cerami et al. 2012; Abeshouse et al. 2017). There were no point mutations and very few DNA copy number alterations reported in the genes encoding the key HBP enzymes (Table S1). Therefore, the activation of the HBP in these subtypes of sarcoma is unlikely to be caused by genomic alterations.

Different types of regulatory mechanisms of the HBP enzymes have been studied in models other than sarcoma. For example, it has been demonstrated that transcription factors c‐MYC, SP1, XBP1s, and ATF4 can directly mediate transcriptional regulation of GFPT1 and/or GFPT2 (Sayeski 1997; Yamazaki et al. 2000; Wang et al. 2014; Moloughney et al. 2016; Chaveroux et al. 2016; Wang et al. 2025). XBP1s also mediates the expression of other HBP enzymes, such as GNPNAT1 and PGM3 (Wang et al. 2014). XBP1s or ATF4 are nutrient‐responsive effectors of the unfolded protein response induced by endoplasmic reticulum (ER) stress (Wang et al. 2014; Chaveroux et al. 2016). This demonstrates that some of these regulatory mechanisms may become activated by tumor microenvironmental factors that activate cellular stress response. Activation of the HBP enzymes can also be mediated through post‐translational modifications. For example, the PKA‐mediated phosphorylation in the glutamine amidotransferase domain at Ser205, Ser235, and Ser243 of GFPT1, and Ser202 of GFPT2, can positively modulate their activity (Hu et al. 2004; Ruegenberg et al. 2021; Li et al. 2007). Many of these effects can be tissue‐ and tumor‐type specific (Ruegenberg et al. 2021); therefore, future studies are needed to further elucidate these mechanisms in different types of sarcoma.

## 5 N‐/O‐Linked Glycosylation and O‐GlcNAcylation in Sarcoma

Post‐translational modifications by glycosylation can affect multiple types of proteins, including oncogenes, transcription factors, and protein kinases (Hanover et al. 2018; Vasconcelos‐Dos‐Santos et al. 2015; Slawson and Hart 2011). These modifications can contribute to the invasive properties of cancer cells and alter cancer cell plasticity. Table 1 and Figure 1 summarize the key features of the O‐GlcNAcylation, N‐linked glycosylation, and O‐linked glycosylation, as reviewed previously by Schjoldager et al. (2020).

**Table 1:** Key characteristics of N‐ and O‐linked glycosylation and O‐GlcNAcylation.

	Glycosylation type					
	N‐linked glycosylation	O‐linked glycosylation				
		O‐linked mucin‐type	O‐GlcNAcylation			
Precursors	UDP‐GlcNAc	UDP‐GalNAc	UDP‐GlcNAc			
Substrates	Secretory proteins	Mucins, ECM proteins, cell surface receptors, and ligands	Signaling proteins, transcription factors, metabolic regulators			
Modified residues	Asn‐X‐Ser/Thr	Ser, Thr				
Subcellular localization	ER, Golgi	Golgi	ER	Mitochondria	Cytoplasm	Nucleus
Reversible	No	Yes	No		Yes – through OGA	
Elongation	Yes	≥ 1 GalNAc added	No			
Cellular processes	Folding and stability	Folding, stability, antibody recognition, adhesion	Signaling, metabolism, and cell fate			

Abbreviations: Asn, asparagine; ECM, extracellular matrix; ER, endoplasmic reticulum; GalNAc, N‐acetylgalactosamine; GlcNAc, N‐acetylglucosamine; OGA, O‐GlcNAcase; Ser, serine; Thr, threonine; UDP, uridine diphosphate.

O‐GlcNAcylation is a type of glycosylation where a single GlcNAc residue is attached to the serine (Ser) or threonine (Thr) residues of the protein. O‐GlcNAcylation is a post‐translational modification that occurs in the cytoplasm, nucleus, mitochondria, and endoplasmic reticulum, while the removal of the O‐GlcNAc residue can only occur via OGA activity in the nucleus and cytoplasm (Figure 1) (Lee, Pyo et al. 2021). O‐GlcNAcylation is a single‐residue modification, as opposed to the O‐linked glycosylation, which involves the elongation of glycans attached to the hydroxyl groups of the Ser or Thr residues (Schjoldager et al. 2020). O‐GlcNAcylation is involved in regulating transcription, cell cycle progression, metabolism, differentiation, and stress response (Schjoldager et al. 2020; Sun et al. 2016). Altered O‐GlcNAcylation of oncogenic proteins has been shown to enhance their stability (Paneque et al. 2023; Jayaprakash and Surolia 2017; Solá et al. 2007). For example, in Ewing sarcoma, O‐GlcNAcylation increases transcriptional activity of the EWS‐FLI1 fusion protein, which is the key oncogenic driver in this disease that acts as an aberrant transcription factor and chromatin regulator (Bachmaier et al. 2009; Shimizu et al. 2018; Riggi et al. 2014). Deng et al. (2020) found that O‐GlcNAcylation is regulated in osteosarcoma by ROCK2 (Rho‐associated coiled‐coil forming protein kinase). The authors showed that ROCK2 affects the degradation of OGT, which contributes to increased O‐GlcNAcylation, disease progression, and therapeutic resistance to TRAIL (TNF‐related apoptosis‐inducing ligand) (Deng et al. 2020). In our previous study, we hypothesized that c‐MYC may be a potential transcription factor stabilized by aberrant O‐GlcNAcylation in leiomyosarcoma (Tolwani et al. 2021). We identified a significant enrichment of the c‐MYC target genes in a subset of leiomyosarcoma that showed increased expression of GFPT2 and other genes encoding the HBP enzymes (Tolwani et al. 2021). We also observed a positive correlation between the expression of GFPT2 and c‐MYC proteins in leiomyosarcoma (Tolwani et al. 2021). Based on these findings, we propose that the potential role of c‐MYC O‐GlcNAcylation in leiomyosarcoma warrants further investigation.

N‐linked glycosylation is a type of protein glycosylation in which sugar molecules are attached to the asparagine (Asn) residues within the consensus sequence Asn‐X‐Ser/Thr of the proteins, where X can be any amino acid except proline (Schjoldager et al. 2020; Stanley et al. 2022). Initiation of N‐linked glycosylation occurs co‐translationally and is regulated by the oligosaccharyltransferase complex (Schjoldager et al. 2020). N‐linked glycosylation occurs within the endoplasmic reticulum and Golgi apparatus (Figure 1 and Table 1). N‐linked glycosylation plays an essential role in protein folding, stability, and interactions, which in turn affects cell adhesion, signaling, and immune response (Schjoldager et al. 2020). Wang et al. (1999) proposed that the EWS‐FLI1 fusion protein may have four potential N‐glycosylation sites, but this has not been experimentally confirmed. Altered N‐linked glycosylation has been associated with tumor progression in endometrial cancer (Mittal et al. 2021), liver cancer (Mehta et al. 2015), and breast cancer (Scott and Drake 2019). In sarcoma, the relevance of N‐glycosylation remains to be explored.

Finally, O‐linked glycosylation is a type of protein glycosylation in which sugar molecules are attached to the Ser or Thr residues of proteins such as mucins, extracellular matrix proteins, cell surface receptors, and ligands (Kudelka et al. 2015; Hang and Bertozzi 2005). O‐linked glycosylation occurs predominantly in the Golgi apparatus and is involved in protein stability, cell adhesion, and inflammation (Hang and Bertozzi 2005). The prevalence and the role of the O‐linked glycosylation in cancer and sarcoma remain understudied.

## 6 Glycosylation Sites in the Molecular Drivers of Sarcoma and Benign Mesenchymal Tumors

**Figure 2:**
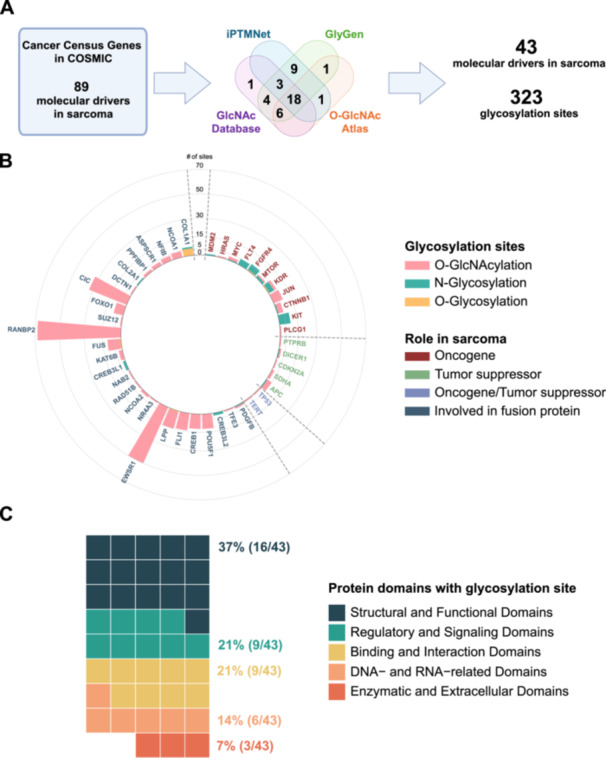
Glycosylation sites in molecular drivers of sarcoma and benign mesenchymal tumors. (A) Workflow used to identify experimentally validated O‐GlcNAcylation and O‐ and N‐linked glycosylation sites in 43 molecular drivers of sarcoma and benign mesenchymal tumors, as reported in the iPTMNet, GlyGen, O‐GlcNAc Atlas, and GlcNAc databases. (B) Frequency of glycosylation sites in the identified molecular drivers classified by their function. (C) Protein domains of molecular drivers of sarcoma and benign mesenchymal tumors with reported glycosylation sites. (COSMIC: Catalogue of Somatic Mutations in Cancer; iPTMnet: Integrated Post‐Translational Modification Network).

In cancer, glycosylation of oncogenes, protein kinases, and transcription factors contributes to tumor development, progression, and resistance to therapy (Hanover et al. 2018; Vasconcelos‐Dos‐Santos et al. 2015; Slawson and Hart 2011). We hypothesize that these post‐translational modifications have a similar effect in sarcoma. Therefore, we sought to catalog previously reported glycosylation sites in the molecular drivers of sarcoma to provide the rationale for future studies on their role in these tumors. First, we assembled a list of 89 molecular drivers in multiple histological subtypes of bone and soft tissue tumors included in the Cancer Gene Census in the Catalogue of Somatic Mutations in Cancer (COSMIC) database [v99] (Figure 2A) (Tate et al. 2019). We then queried the known glycosylation sites of these 89 proteins in four curated databases of post‐translational modifications, including iPTMnet [v6.1], GlyGen [v1.12.3], O‐GlcNAc Database [v1.2], and O‐GlcNAcAtlas [v2.0] (Figure 2A) (Huang et al. 2018; York et al. 2020; Wulff‐Fuentes et al. 2021; Ma et al. 2021). In these databases, we identified the previously reported glycosylation sites in 43 driver molecules that are implicated in over 30 histological subtypes of sarcoma and benign mesenchymal tumors (Figure 2A and Table 2). These molecules are primarily involved in fusion proteins (Table 2). Other molecular drivers of sarcoma that carry glycosylation sites include several oncogenes (i.e., KIT, JUN, FGFR4, FLT4, COL2A1, HRAS, MDM2, PLCG1) and tumor suppressors (i.e., APC, SDHA, PTPRB, CDKN2A). TP53, which can act as either an oncogene or a tumor suppressor in different types of sarcoma, contains a single O‐GlcNAcylation site (Table 2).

**Table 2:** Molecular drivers of sarcoma and benign mesenchymal tumors that harbor N‐/O‐linked glycosylation and O‐GlcNAcylation sites, as reported in the databases of post‐translational modifications.

Protein name	Role in sarcoma	Tumor type	Total number of glycosylation sites	N‐Glycosylation sites	O‐GlcNAc sites	O‐Glycosylation sites
RANBP2	FU	IMT	68 sites	No	68 sites	No
EWSR1	FU	ES, DSRCT, CCS, and other types of sarcoma	50 sites	No	50 sites	No
CIC	FU	CIC‐rearranged sarcoma	33 sites	No	33 sites	No
LPP	FU	LPM	15 sites	No	14 sites	1 site
FLI1	FU	ES	14 sites	No	14 sites	No
CREB1	FU	CCS, AFH	12 sites	No	12 sites	No
POU5F1	FU	Myoepithelioma/mixed tumor	12 sites	No	12 sites	No
FUS	FU	ES, AFH, low‐grade FMS, MLPS	11 sites	No	10 sites	1 site
KIT	Onc	GIST	9 sites	9 sites	No	No
FOXO1	FU	ARMS	8 sites	No	8 sites	No
JUN	Onc	Undifferentiated liposarcoma	8 sites	No	8 sites	No
COL1A1	FU	DFSP	7 sites	1 site	No	6 sites
KDR	Onc	AS	7 sites	4 sites	2 sites	1 site
FGFR4	Onc	ERMS	6 sites	5 sites	No	1 site
CTNNB1	Onc	DT	5 sites	1 site	4 sites	No
APC	TSG	DT	5 sites	1 site	4 sites	No
FLT4	Onc	AS	5 sites	5 sites	No	No
NCOA1	FU	ARMS	5 sites	No	5 sites	No
NFIB	FU	LPM	4 sites	No	4 sites	No
ASPSCR1	FU	ASPS	3 sites	No	3 sites	No
KAT6B	FU	Uterine sarcoma	3 sites	No	3 sites	No
MTOR	Onc	Multiple types of sarcoma	3 sites	2 sites	1 site	No
MYC	Onc	Multiple types of sarcoma	3 sites	1 site	2 sites	No
CREB3L1	FU	Low‐grade FMS, SEF	2 sites	2 sites	No	No
CREB3L2	FU	Low‐grade FMS, SEF	2 sites	2 sites	No	No
DICER1	TSG	TGCT, ERMS	2 sites	1 site	1 site	No
NCOA2	FU	AF, CHS: ARMS, spindle cell RMS	2 sites	No	2 sites	No
PDGFB	FU	DFSP	2 sites	1 site	1 site	No
PPFIBP1	FU	IMT	2 sites	No	2 sites	No
SDHA	TSG	GIST	2 sites	No	2 sites	No
CDKN2A	TSG	Multiple types of sarcoma	1 site	No	1 site	No
COL2A1	Onc	CHS	1 site	1 site	No	No
HRAS	Onc	ERMS	1 site	No	1 site	No
MDM2	Onc	WDLPS, DDLPS	1 site	1 site	No	No
NAB2	FU	SFT	1 site	No	1 site	No
NR4A3	FU	EMC	1 site	No	1 site	No
PLCG1	Onc	AS	1 site	No	1 site	No
PTPRB	TSG	AS	1 site	No	1 site	No
RAD51B	FU	LM, PEComa	1 site	No	1 site	No
SUZ12	FU	LGESS	1 site	No	1 site	No
TERT	Onc, TSG	Multiple types of sarcoma	1 site	No	1 site	No
TFE3	FU	ASPS	1 site	No	1 site	No
TP53	Onc, TSG	Multiple types of sarcoma	1 site	No	1 site	No

Abbreviations: AF, angiofibroma; AFH, angiomatoid fibrous histiocytoma; ARMS, alveolar rhabdomyosarcoma; AS, angiosarcoma; ASPS, alveolar soft part sarcoma; CCS, clear cell sarcoma; CHS, chondrosarcoma; DDLPS, dedifferentiated liposarcoma; DFSP, dermatofibrosarcoma protuberans; DSRCT, desmoplastic small round cell tumor; DT, desmoid tumor; EMC, extraskeletal myxoid chondrosarcoma; ERMS, embryonal rhabdomyosarcoma; ES, Ewing sarcoma; FMS, fibromyxoid sarcoma; FU, protein involved in fusion; GIST, gastrointestinal stromal tumor; IMT, inflammatory myofibroblastic tumor; LGESS, low‐grade endometrial stromal sarcoma; LM, leiomyoma; LPM, lipoma; MLPS, myxoid/round cell liposarcoma; Onc, oncoprotein SEF, sclerosing epithelioid fibrosarcoma; SFT, solitary fibrous tumor; TGCT, Tenosynovial giant cell tumor; TSG, tumor suppressor; WDLPS, well‐differentiated liposarcoma.

Our analysis revealed that the predominant glycosylation type in the 43 molecular drivers of sarcoma is the O‐GlcNAc modification, representing 85.4% (276/323) of the glycosylation sites in these proteins (Figure 2B and Table 2). The N‐linked and O‐linked glycosylation sites comprised 11.5% (37/323) and 3.1% (10/323) of the reported modifications, respectively (Figure 2B and Table 2). RANBP2, a protein involved in a fusion with ALK in inflammatory myofibroblastic tumors, contains the highest number of O‐GlcNAcylation sites among the examined proteins (Figure 2B and Table 2) (Ma et al. 2003; Chen and Lee 2008; Mariño‐Enríquez et al. 2011). Interestingly, the only protein in our analysis harboring all three types of glycosylation sites is the kinase insert domain receptor (KDR), which is recognized as a molecular driver of primary mammary angiosarcoma (Figure 2B and Table 2) (Beca et al. 2020). The KIT protein has the highest number of N‐linked glycosylation sites (9 sites), and COL1A1 has the highest number of O‐linked glycosylation sites (6 sites) (Figure 2B and Table 2). Given the high frequency of O‐GlcNAcylation sites in key driver proteins across various sarcoma subtypes, we hypothesize that targeting the OGT enzyme, which catalyzes O‐GlcNAcylation, could offer a therapeutic benefit in these tumors. However, these glycosylation sites were identified in healthy tissues and different types of cancer, not in sarcoma. Therefore, the role of these modifications and the therapeutic potential of targeting glycosylation in these molecular drivers of sarcoma remain to be explored. Only in Ewing sarcoma has it been experimentally confirmed that the transcriptional activity of the chimeric EWS‐FLI1 protein is indeed modulated by O‐GlcNAcylation (Bachmaier et al. 2009).

Next, we sought to identify protein domains in the molecular drivers of sarcoma that frequently carry glycosylation sites. Certain protein domains have an increased propensity for the O‐linked and N‐linked glycosylation due to specific structural characteristics and functional requirements (Love and Hanover 2005; Bagdonaite et al. 2022). Extracellular domains are frequently glycosylated in proteins destined for secretion or cell surface localization (Varki 1993). Specific amino acid motifs, such as the Asn‐X‐Ser/Thr motif, act as recognition sites for glycosyltransferases, facilitating the N‐linked glycosylation. Additionally, the domains with structural flexibility or exposed regions, such as the extracellular loops in membrane proteins, are the favored sites for glycan attachment due to enhanced accessibility (Rudd and Dwek 1997). As previously reviewed by Love and Hanover (2005), the O‐GlcNAcylated proteome mostly consists of phosphoproteins, cytoskeletal proteins, proteins involved in transcriptional complexes, and nuclear pore complexes. O‐GlcNAcylated proteins are mostly involved in transcription/translation, carbohydrate metabolism, and stress‐related processes (Love and Hanover 2005).

To identify protein domains with the highest frequency of glycosylation sites in the 43 molecular drivers of sarcoma, we obtained the protein structure annotation from the following databases: UniProt KB (release 2024_04), InterPro (v101.0), Simple Modular Architecture Research Tool (SMART, v9.0), Mutagenetix (accessed March 1, 2024), Expasy Prosite (release October 2020) and Human Protein Atlas (proteinatlas.org) (The UniProt Consortium 2017; Paysan‐Lafosse et al. 2023; Letunic et al. 2021; Beutler 2013; Duvaud et al. 2021; Uhlén et al. 2015), and we collated protein domains and reported glycosylation sites (Figure 2C and Table S2). We identified 43 domains with reported glycosylation sites in the molecular drivers of sarcoma (Table S2). We grouped these domains into five categories: 16 domains related to the structure and function of the protein, 9 regulatory and signaling domains, 9 domains involved in protein binding and interactions, 6 DNA‐ and RNA‐binding domains, and 3 enzymatic and extracellular domains (Figure 2C). Based on the location of the glycosylation sites in the molecular drivers of sarcoma, we hypothesize that glycosylation of these proteins can primarily affect: (i) protein interactions, and (ii) regulatory and signaling domains.

i. Protein–protein and protein‐nucleic acid interactions. Glycosylation can influence protein–protein interactions by creating steric hindrance, masking binding sites, and altering protein surface topology and interactions (Varki et al. 2015; Freeze and Kranz 2010; Helenius and Aebi 2001). Glycans facilitate protein recognition and induce conformational changes in proteins, which may impact their folding, stability, and interaction dynamics (Varki et al. 2015). Moreover, glycosylation can stabilize protein complexes through hydrogen bonds or hydrophobic/hydrophilic interactions (Varki et al. 2015). On the cell surface, glycosylation may influence cell–cell recognition and signaling (Varki et al. 2015). Glycosylation is also essential for the proper function of the Fc region of the antibodies, playing a key role in regulating antibody‐dependent cellular cytotoxicity and complement activation (Marth and Grewal 2008). Here, we found that glycosylation sites in molecular drivers of sarcoma are common in the structural domains, binding and interaction sites, and immunoglobulin‐like domains (Figure 2C and Table S2). Selected molecular drivers of sarcoma also have glycosylation sites within their RNA‐binding sites (e.g., EWSR1 and FUS) and DNA‐binding sites (e.g., FLI1 and POU5F1), suggesting the potential role of these modifications in transcriptional regulation and modulation of gene expression.
ii. Regulatory and signaling domains. O‐GlcNAcylation may compete with phosphorylation for the same Ser/Thr residues, suggesting a mutual exclusivity between these two modifications (van der Laarse et al. 2018). This competitive interplay acts as a molecular switch that determines the fate and function of key oncogenic drivers, such as c‐MYC, JUN, TP53, and β‐catenin. For example, phosphorylation of the c‐MYC oncoprotein at Thr58 promotes its degradation, whereas O‐GlcNAcylation at the same site blocks phosphorylation and contributes to increased stability of c‐MYC (Chou et al. 1995; Itkonen et al. 2013). Our previous work suggested that c‐MYC may be stabilized by O‐GlcNAcylation as a result of the activated HBP, also in a subset of LMS (Tolwani et al. 2021). Similar to c‐MYC, O‐GlcNAcylation sites in the JUN protein, an oncogene in undifferentiated liposarcoma, overlap with the JNK‐mediated phosphorylation sites that regulate JUN activity (Table S2) (Raivich 2008). The dynamic interplay between O‐GlcNAcylation and phosphorylation also regulates the stability and activity of TP53, which is the most common oncogenic driver in sarcoma. Yang et al. (2006) demonstrated that O‐GlcNAcylation at Ser149 stabilizes TP53 by impeding phosphorylation at Thr155, which in turn inhibits ubiquitin‐dependent proteolysis of TP53. Also in β‐catenin, the key oncogenic driver in desmoid tumors, O‐GlcNAcylation at Thr41 interferes with GSK3β‐mediated phosphorylation, which in turn blocks ubiquitination and proteasomal degradation of β‐catenin (Olivier‐Van Stichelen et al. 2012). Collectively, these findings underscore O‐GlcNAcylation as a key post‐translational modification that stabilizes oncogenic proteins by antagonizing phosphorylation‐dependent degradation mechanisms. The functional studies demonstrating the crosstalk between phosphorylation and O‐GlcNAcylation have been published mostly in preclinical models of cancer. If the same crosstalk is confirmed in sarcoma models, targeting the HBP or downstream O‐GlcNAcylation may offer a promising strategy to destabilize key oncogenic proteins in mesenchymal tumors.

## 7 Therapeutic Effect of the HBP Inhibition in Preclinical Models of Cancer and Sarcoma

In preclinical cancer models, pharmacological inhibition of the HBP directly suppresses cell growth and proliferation, and sensitizes cancer cells to chemotherapy, radiation therapy, and immunotherapy (as reviewed by Lam et al. 2021; Vasconcelos‐Dos‐Santos et al. 2015). Most studies to date have investigated the effects of small‐molecule inhibitors targeting GFPT1/2, the first rate‐limiting enzyme of the HBP, as well as inhibitors of OGT, which catalyzes O‐GlcNAcylation (Table 3). A few studies have also focused on targeting the PGM3 and OGA enzymes (Table 3). While therapeutic targeting of the HBP has been evaluated in a broad range of cancer models, most reports are limited to in vitro studies of cell viability and apoptosis. Inhibition of GFPT1/2 through drugs like DON (6‐diazo‐5‐oxo‐L‐norleucine) and azaserine (O‐diazoacetyl‐L‐serine), demonstrated promising effects both in vitro and in vivo. Targeting OGT has also been investigated using various inhibitors, with notable functional outcomes. Multiple studies demonstrated that OGT inhibitors can sensitize cancer cells to standard chemotherapy or can have an additive or synergistic effect in combination with other therapeutic agents (Table 3).

**Table 3:** Pharmacological inhibitors of HBP enzymes investigated in preclinical models of cancer.

HBP target	Drug name	Cancer type	Model (cell lines or mouse models)	Treatment effect	Reference
GFPT1/2	DON	Lung, colon, breast	Patient‐derived xenograft models	Tumor regression	Ovejera et al. (1979)
		Lung and breast	Cell lines (A549, H1299, MCF‐7)	Increased apoptosis	Walter et al. (2020)
		Colorectal	Cells lines (HT29, HCT116)	Decreased β‐catenin levels	Olivier‐Van Stichelen et al. (2012)
		AML	Cell lines (OCI‐AML3, HL‐60) Subcutaneous xenograft model (HL‐60 cells)	Inhibited proliferation, induced apoptosis	Asthana et al. (2018)
		Lung	Co‐culture of activated T cells and cancer cells (H2009, SK‐MES‐1)	Increased IL‐2 release, decreased cancer cell viability	Chen et al. (2021)
		PDAC	Cell lines (BxPC3, SW1990)	Inhibited proliferation and invasive potential, induced apoptosis	Jia et al. (2020)
			Orthotopic xenograft model (murine KPC cells, human 779E and PaTu 8998T cells)	Suppressed cell growth through inhibition of asparagine metabolism, attenuated metastasis	Recouvreux et al. (2024)
			Cell lines (BxPC3, SW1990, SU.8686) Subcutaneous xenograft models (BxPC3, SW1990 cells)	Decreased tumor volume, induced ROS formation and ferroptosis	Xiao et al. (2023)
			Subcutaneous (S2VP10 cells) and orthotopic (KPC cells) xenograft models	Decreased tumor growth and metastasis, modulation of ECM, and increased sensitivity to anti‐PD1 therapy	Sharma et al. (2020)
		Glioblastoma	Cell lines (U251, U87, SF767)	Decreased proliferation	Ohba and Hirose (2020)
		Atypical teratoid/rhabdoid tumors (AT/RT)	Orthotopic models (BT12 and CHLA‐06‐ATRT cells)	Decreased tumor growth, induced apoptosis	Wang et al. (2019)
	Azaserine	DLBCL	Cell lines (OCI‐LY10, MS, EJ)	Induced apoptosis	Pham et al. (2016)
		PDAC	Cell lines (BxPC3, SW1990)	Inhibited proliferation and invasive potential, induced apoptosis	Jia et al. (2020)
			Cell lines (BxPC3, SW1990, SU.8686)	Induced ROS formation and ferroptosis	Ohba and Hirose (2020)
		Lung	Cell lines (A549, H460, DFCI‐024, H157, H2122) Subcutaneous xenograft models (H460, A549 cells)	Decreased cell viability in vitro and tumor growth in vivo	Kim et al. (2020)
PGM3	FR054	Breast	Cell lines (MDA‐MB‐231, MDA‐MB‐468, MDA‐MB‐361, MCF7, T‐47D, BT‐474, SKBR‐3), Subcutaneous xenograft model (MDA‐MB‐231 cells)	Inhibited proliferation, induced apoptosis	Ricciardiello et al. (2018)
	FR054	PDAC	Cell lines (MiaPaCa‐2, PANC1, BxPC3)	Increased ferroptosis, unfolded protein response	Zerbato et al. (2023)
	FR054	Lung	Cell lines (H460, H2122, H1373), Subcutaneous xenograft model (H1373 cells)	Decreased colony formation in vitro, decreased tumor growth, and increased apoptosis in vivo	Lee et al. (2022)
OGT	BADGP	AML	Cell lines (OCI‐AML3, HL‐60)	Inhibited proliferation, induced cell differentiation	Asthana et al. (2018)
	OSMI‐1	Liver	Cell line (HepG2) Subcutaneous xenograft model (HepG2 cells)	Combined with doxorubicin: decreased cell viability and increased apoptosis in vitro; induced ER stress response, decreased tumor growth, and increased apoptosis in vivo	Lee and Kwon (2020)
		Colorectal and liver	Cell lines (HCT116, HepG2), Subcutaneous xenograft model (HCT116 cells)	Combined with TRAIL: Decreased viability and colony formation in vitro, increased apoptosis, and ER stress response in vitro; decreased tumor growth and increased apoptosis and ER stress response in vivo	Lee, Lee et al. (2021)
		Liver and breast	Cell lines (MCF‐7, SMMC‐7721)	Sensitization to doxorubicin; OSMI‐1 combined with doxorubicin: decreased activated NF‐κB and AKT	Liu et al. (2018)
		Breast	Cell lines (tamoxifen sensitive and resistant MCF‐7 cells)	Decreased cell viability and proliferation	Barkovskaya et al. (2020)
		PDAC	Cell lines (BxPC3, SW1990)	Decreased β‐catenin activity	Jia et al. (2020)
		Lung	Cell line (H460, H2122)	Decreased cell viability	Kim et al. (2020)
		AML	Cell lines (OCI‐AML3, HL‐60)	Inhibited proliferation, induced cell differentiation	Asthana et al. (2018)
		Prostate	Cell lines (PC‐3, WPMY‐1)	Synergistic effect in combination with doxorubicin	Makwana et al. (2020)
	OSMI‐2	Prostate	Cell lines (LNCaP, C4‐2, 22RV1)	Induced metabolic reprogramming, OSMI‐2 combined with a pan‐CDK inhibitor: decreased mitochondrial respiration	Gondane et al. (2022)
	OSMI‐2 OSMI‐4	Prostate, breast, colon	Cell lines (prostate cancer: LNCaP, C4‐2, 22RV1, PC3, breast cancer: MDA‐MB‐231, colon cancer: HCT116)	Synthetic lethality between OSMI‐2/4 and pan‐CDK inhibitors	Itkonen et al. (2020)
	L01	Breast	Cell lines (MCF‐7, MDA‐MB‐231)	L01 combined with bortezomib: induced apoptosis	Liu, Wang et al. (2019)
			Cell line (MCF‐7)	L01 combined with Adriamycin: decreased cell viability	Xie et al. (2021)
	ST045849	Prostate	Cel lines (LNCaP, VcaP, PC3)	Decreased cell viability, destabilized c‐MYC	Itkonen et al. (2013)
	HLY838	Liver	Cell lines (PLC/PRF/5, MHCC‐97H, SK‐Hep1) Subcutaneous xenograft model (MHCC‐97H cells)	Enhanced antiproliferative activity of CDK9 inhibitors	Shan et al. (2023)
OGA	Thiamet‐G	Colorectal	AOM/DSS‐induced model	Increased sensitivity to 5‐FU	Very et al. (2022)
	PugNAc, Thiamet‐G, ketoconazole	Mantle cell lymphoma	Cell lines (Jeko‐1, Granta‐519, SP49)	Increased sensitivity to bortezomib, increased apoptosis	Luanpitpong et al. (2018)

Abbreviations: AML, acute myeloid leukemia; AOM/DSS, azoxymethane (AOM)/dextran sulfate sodium (DSS); Azaserine, O‐diazoacetyl‐L‐serine; BADGP, benzyl 2‐acetamido‐2‐deoxy‐α‐D‐galactopyranoside; DLBCL, diffuse large B‐cell lymphoma; DON, 6‐diazo‐5‐oxo‐L‐norleucine; ECM, extracellular matrix; ER, endoplasmic reticulum; 5‐FU, 5‐fluorouracil; OGT, O‐GlcNAc transferase; PDAC, pancreatic ductal adenocarcinoma; PugNAc, 1,5‐hydroximolactone; ROS, reactive oxygen species.

Therapeutic targeting of the HBP in sarcoma has been explored mostly in Ewing sarcoma, osteosarcoma, and gastrointestinal stromal tumors. In Ewing sarcoma models, inhibiting the HBP with DON disrupted O‐GlcNAcylation of EWS‐FLI1 and impeded activation of transcriptional activator ID2, which is regulated by EWS‐FLI1 (Bachmaier et al. 2009). These results suggest that drugs modulating the glycosylation of EWS‐FLI1 could effectively disrupt its activity, which offers a promising approach for the treatment of Ewing sarcoma. Other studies in Ewing sarcoma cell lines explored the effect of tunicamycin, which induces ER stress response and inhibits N‐linked glycosylation. In RD‐ES cells, tunicamycin reduced the expression of the EWS‐FLI1 fusion protein (Wang et al. 1999). Tunicamycin treatment also decreased the IGF‐1R levels on the cell surface of both RD‐ES and ES‐1 cells, suggesting that inhibiting N‐linked glycosylation interferes with the translocation of the IGF‐1R to the cell surface (Girnita et al. 2000). Several studies also investigated the effect of targeting general glucose metabolism and N‐linked glycosylation with 2‐deoxyglucose (2‐DG) in sarcoma. In preclinical models of osteosarcoma, treatment with 2‐DG reduced the cell‐surface expression of MICA (major histocompatibility class I‐related chain molecule A) under normoxic conditions (Yamada et al. 2018). In preclinical models of gastrointestinal stromal tumors, 2‐DG treatment inhibited N‐glycosylation of KIT, increased apoptosis, and suppressed the growth of both imatinib‐sensitive and imatinib‐resistant cell lines (Mühlenberg et al. 2015). These studies provide the rationale for further investigation of these inhibitors in sarcoma.

Although several molecular compounds are available to inhibit HBP, the understanding of their mechanisms of action remains limited. For instance, 2‐DG is a competitive inhibitor of hexokinase, which inhibits the production of G6P. Therefore, the effect of 2‐DG on the HBP may result from a general inhibition of glucose metabolism, rather than from a direct, targeted inhibition of the pathway itself. In addition, it is crucial to better understand the molecular processes altered by the HBP‐targeting drugs to mitigate their nonspecific toxicity and adverse effects.

## 8 Future Perspectives on Therapeutic Targeting of the HBP in Sarcoma

Elevated O‐GlcNAcylation and OGT levels are considered to be one of the oncogenic hallmarks in many types of cancer (Itkonen et al. 2013; Ferrer et al. 2016; Gu et al. 2010). O‐GlcNAcylation can also underlie resistance to chemotherapy. For example, Luanpitpong et al. (2017) described the mechanism of resistance to cisplatin induced by hyper‐O‐GlcNAcylation of c‐MYC and TP53 as an effect of treating lung cancer cells with OGA inhibitors. In that study, O‐GlcNAcylation inhibited c‐MYC ubiquitination, while promoting TP53 ubiquitination and its subsequent proteasomal degradation, which impeded cisplatin‐induced apoptosis (Luanpitpong et al. 2017). Another study showed elevated O‐GlcNAcylation in association with resistance to doxorubicin and camptothecin in liver and breast cancer cell lines (Liu et al. 2018). Therefore, therapeutic targeting of the HBP appears as a plausible strategy to sensitize cancer cells to chemotherapy. Several studies summarized in Table 3 demonstrated that OGT inhibitors either sensitized cancer cells to doxorubicin or showed a synergistic effect with doxorubicin. Doxorubicin is the most common first‐line chemotherapy used to treat sarcoma, with response rates in clinical trials between 10% and 25% (Schöffski et al. 2014). Thus, it is worth investigating whether OGT inhibitors could sensitize resistant cells to doxorubicin or show a synergistic effect in combination with doxorubicin in preclinical models of sarcoma.

Several OGT inhibitors (OSMI‐1, OSMI‐2, HLY838) have also been shown to enhance the effect of CDK (cyclin‐dependent kinase) inhibitors in multiple preclinical cancer models (Table 3). Deregulation of the CDKN2A‐CCND‐CDK4/6‐RB pathway is observed in approximately 25% of sarcomas, which prompted the clinical evaluation of selective CDK4/6 inhibitors in these tumors (Hsu et al. 2022). Despite promising results of clinical trials with CDK4/6 inhibitors in dedifferentiated and well‐differentiated liposarcoma, most histological subtypes of sarcoma exhibit resistance to this treatment (Hsu et al. 2022). Developing new strategies to overcome resistance to CDK inhibitors may improve the clinical utility of these drugs in sarcomas with Rb pathway dysregulation. Further studies are needed to explore whether OGT inhibitors could exhibit antiproliferative effects or synthetic lethality when combined with CDK inhibitors in preclinical sarcoma models, as seen in preclinical cancer models.

Considering the significant role of glycosylation in immunity (Marth and Grewal 2008), it has also been explored whether the HBP‐targeting drugs could enhance anti‐cancer immune response. For instance, treatment with metformin and 2‐DG, which indirectly affect glycosylation levels, had a favorable effect on anti‐tumor immunity by causing deglycosylation and reduced expression of PD‐L1 in triple‐negative breast cancer cells (Repas et al. 2022). Also, targeting GFPT1 and GFPT2 with DON has been found to sensitize pancreatic cancer cells to PD1‐targeting immunotherapy, contributing to tumor regression and extended survival in an orthotopic mouse xenograft model (Sharma et al. 2020). A similar approach could be tested in mouse models of sarcoma, as durable responses to checkpoint inhibitors are very rare in most histological subtypes of sarcoma.

Two previous studies have also demonstrated that the HBP and O‐GlcNAcylation drive the expression and activity of β‐catenin in preclinical models of pancreatic and colorectal cancer (Olivier‐Van Stichelen et al. 2012; Jia et al. 2020). Inhibition of the HBP either by DON or OSMI‐1 in these models decreased β‐catenin activity, cell proliferation, invasive capacity, and triggered cell apoptosis (Olivier‐Van Stichelen et al. 2012; Jia et al. 2020). Several types of sarcoma, such as desmoid‐type fibromatosis, depend on the activation of Wnt/β‐catenin signaling. Therefore, further studies are needed to determine whether targeting the HBP could also impact Wnt/β‐catenin signaling in these tumors.

## 9 Development of Prodrugs and Clinical Trials of the HBP‐Targeting Drugs: Focus on DON

While targeting the HBP has demonstrated promising results in preclinical cancer models, there have been only a limited number of clinical trials of the HBP inhibitors in humans. Excluding drugs like 2‐DG and tunicamycin, which broadly target glycometabolism, DON remains the only HBP‐targeting compound that was investigated in humans. DON is a glutamine antagonist that has been studied as a potential anti‐cancer drug for several decades. The clinical trial data for DON have been thoroughly documented by Lemberg et al. (2018) and Cervantes‐Madrid et al. (2015). In the 1950s, Magill et al. (1957) conducted the first clinical trial of DON in 63 patients with advanced inoperable cancer, lymphoma, and leukemia, reporting partial responses in 15% of patients who were treated for at least 2 weeks. Several phase I and II clinical trials were also conducted in the 1980s, using high intermittent doses of DON as a single agent (Sullivan et al. 1988; Earhart et al. 1982; Kovach et al. 1981; Sklaroff et al. 1980; Rahman et al. 1985). These trials suggested partial response in pediatric patients with hematologic and solid tumors, and showed limited success and significant gastrointestinal toxicity in adult patients (Sullivan et al. 1988; Earhart et al. 1982; Kovach et al. 1981; Sklaroff et al. 1980; Rahman et al. 1985). Currently, there is an interest in developing DON prodrugs to enhance tumor targeting and to minimize the toxicity in non‐tumor tissues that exhibit high glutamine consumption, such as the gastrointestinal tract. Prodrug approaches for DON have focused on chemically modifying three functional groups of the molecule: the carboxylic acid, amino, and diazo groups (Novotná et al. 2023; Sampson et al. 2025). DON prodrugs that have been tested in preclinical and clinical studies are summarized in Table 4.

**Table 4:** DON prodrugs tested in preclinical and clinical studies.

Prodrug name	Design strategy	Development stage	Disease/model tested	Key findings	Reference
JHU‐083 (“4a” in Rais et al. 2016)	Dual modification via ethyl esterification of the carboxylic acid group and L‐leucine conjugation at the amino group	In vivo	Colon cancer, lymphoma, and melanoma mouse models	Enhanced immune anti‐tumor response	Rais et al. (2016); Leone et al. (2019)
Methyl‐POM‐DON‐isopropyl‐ester (“5c” in Rais et al. 2016)	Dual modification via isopropyl esterification of the carboxylic acid group and masking with a methyl‐POM (methyl‐phosphoryloxymethyl) carbamate at the amino group	In vivo	Glioblastoma mouse model	Improved CSF delivery, high stability	Rais et al. (2016)
JHU‐395 (“13 d” in Nedelcovych et al. 2017)	Dual modification via isopropyl esterification of the carboxylic acid group and (phenyl(pivaloyloxy)methoxy)‐carbonyl modification at the amino group	In vitro In vivo	Malignant peripheral nerve sheath tumor (MPNST) (cell lines and mouse model) Pharmacokinetics in swine	Improved CSF delivery in swine Suppressed tumor growth in MPNST	Nedelcovych et al. (2017); Lemberg et al. (2020, 2023)
JHU‐607 (“6” in Tenora et al. 2019)	Dual modification via isopropyl esterification of the carboxylic acid group and adamantane‐lysine acylation at the amino group	In vitro In vivo	Lymphoma (cell lines and mouse model)	Improved tumor delivery, dose‐dependent effect on cell viability	Tenora et al. (2019); Novotná et al. (2024)
DRP‐104 (Sirpiglenastat)	Dual modification via isopropyl esterification of the carboxylic acid group and N‐acetyl‐tryptophan conjugation at the amino group	In vivo Phase 1 and 2 trials NCT07249372 NCT06027086 NCT07430202	Lymphoma (mouse model) Solid tumors	Immune‐modulating anti‐tumor effects in vivo	Rais et al. (2022)
JHU‐1133 (“P11” in Novotna et al. 2023)	Dual modification via tert‐butyl esterification of the carboxylic acid group and acylation with amino‐acid–derived promoiety at the amino group	In vitro In vivo	Lymphoma (cell lines and mouse model)	Improved tumor delivery, dose‐dependent effect on cell viability	Novotná et al. (2023, 2024)
HDON	Dual modification via ethyl esterification of the carboxylic acid group and masking with 4‐nitrobenzyl carbamate at the amino group, activated by hypoxic nitroreductase	In vitro In vivo	Liver, colon, and breast cancer (cell lines and mouse models)	Suppressed tumor growth	Zheng et al. (2024)
Azo‐DON	Dual modification via ethyl esterification of the carboxylic acid group and carbamate conjugation of the amino group with a dimethylamino‐azobenzene moiety, activated by hypoxic azo‐reductase	In vitro In vivo	Liver and colon cancer (cell lines and mouse models)	Suppressed tumor growth	Xu et al. (2024)
Redox‐DON	Redox‐responsive disulfide linker, activated by glutathione	In vitro In vivo	Colon and breast cancer, melanoma (cell lines and mouse models)	Suppressed tumor growth, selective activation with improved safety profile, dose‐dependent effect on cell viability	Prange et al. (2024)
DON‐TK‐BM3	Cyanine dye (BM3) linked via thioketal (TK) bond, activated by near‐infrared light	In vitro In vivo	Lung cancer (cell lines and mouse models)	Suppressed tumor growth, selective activation with real‐time monitoring	Li et al. (2024)

Most prodrug strategies involve attaching enzymatically cleavable carriers, or promoieties, to both the carboxylic acid and amino groups of the DON molecule. Rais et al. (2016) developed several prodrugs for DON, including JHU‐083 (also called 4a) and 5c, aimed at enhancing brain delivery while limiting systemic exposure. This approach provided a targeted anti‐tumor effect of DON in glutamine‐dependent brain tumors (Novotná et al. 2023). Next, Nedelcovych et al. (2017) developed JHU‐395 (also called 13 d), another brain‐targeting DON prodrug that exhibited higher brain‐to‐plasma exposure as a treatment for neurological disorders. In mouse models of malignant peripheral nerve sheath tumor, which is an aggressive type of sarcoma with poor prognosis, JHU‐395 inhibited de novo purine synthesis and tumor growth (Lemberg et al. 2020). Furthermore, JHU‐395 in combination with purine anti‐metabolite Pro‐905, resulted in further inhibition of colony formation and augmented the anti‐tumor efficacy in mice (Lemberg et al. 2023). This approach demonstrated that concurrent inhibition of de novo purine synthesis and purine salvage had a robust anti‐tumor efficacy in malignant peripheral nerve sheath tumors. In 2019, Tenora et al. (2019) reported a novel series of DON prodrugs, including JHU‐607 (compound 6), that were designed to remain inert in plasma and healthy tissues, while being preferentially activated in tumor cells. Later, Rais et al. (2022) introduced DRP‐104 (also called sirpiglenastat), which was the first dipeptide prodrug of DON. DRP‐104 showed strong metabolic stability in both plasma and gut homogenate, exhibited dose‐dependent anti‐tumor efficacy as a single agent, and positively influenced the anti‐tumor immune response (Novotná et al. 2024). Additionally, several studies have demonstrated that DRP‐104 effectively eliminated tumors in preclinical models without significant side effects (Lemberg et al. 2018). For instance, DRP‐104 reduced proliferation and induced apoptosis of prostate cancer cell lines, and inhibited the growth of neuroendocrine prostate cancer xenografts in vivo without causing severe toxicities (Moon et al. 2024). DRP‐104 treatment also provided a survival benefit in a patient‐derived xenograft model of pancreatic cancer when combined with MEK inhibitor trametinib (Encarnación‐Rosado et al. 2024). Moreover, DRP‐104 inhibited glutamine‐dependent nucleotide synthesis and enhanced the anti‐tumor T cell response in a patient‐derived xenograft model of KEAP1‐mutant lung cancer (Pillai et al. 2024). Based on the promising preclinical results of DRP‐104, it has entered clinical trials. As of March 2026, there are 3 registered phase 1 and 2 clinical trials of DRP‐104 in combination with immunotherapy in different types of solid tumors (NCT06027086, NCT07249372 and NCT07430202) (ClinicalTrials.gov [2026]). However, despite the high efficacy and tolerability, DRP‐104 has exhibited limited aqueous solubility and instability of its isopropyl ester promoiety, leading to reduced systemic prodrug exposure (Novotná et al. 2023). Consequently, a new generation of prodrugs similar to DRP‐104, including prodrug JHU‐1133 (compound P11), was reported in 2023 (Novotná et al. 2023). JHU‐1133 demonstrated good metabolic stability in plasma and intestinal homogenate, along with improved aqueous solubility (Novotná et al. 2023, 2024).

In addition to the prodrug designs focused on the amino and carboxylate groups of DON, other strategies have also targeted the diazo group by converting it into an inert diazo precursor. Gao et al. (2021) explored two distinct diazo precursors, imidazotetrazine and nitrous amide, as promoieties in the design of DON prodrugs. The study published by this group confirmed that the imidazotetrazine‐based prodrug is stable enough to convert into N‐acetyl DON, which is eventually transformed into DON by aminoacylase and other hydrolytic enzymes (Gao et al. 2021). In contrast, the nitrous amide‐based prodrug did not exhibit sufficient stability (Gao et al. 2021). This study concluded that further improvements are needed to translate the diazo prodrug into practical applications.

Another strategy to improve the selectivity and efficacy of DON is the use of hypoxia‐activated prodrugs, such as HDON and Azo‐DON (Zheng et al. 2024; Xu et al. 2024). These prodrugs selectively target hypoxic tumor tissues, while remaining inactive in normal tissues with sufficient oxygen levels. HDON is activated by the high expression of nitroreductase, and Azo‐DON is reduced to DON by the overexpression of azo‐reductase in a hypoxic tumor environment (Zheng et al. 2024; Xu et al. 2024). HDON and Azo‐DON demonstrated a significant anti‐tumorigenic effect as a monotherapy in the highly hypoxic H22 murine hepatoma cancer model (Zheng et al. 2024; Xu et al. 2024). In models with lower hypoxia levels, such as CT26 colon cancer, MC38 murine colon cancer, and 4T1 murine breast cancer models, the efficacy of HDON and Azo‐DON was augmented by the nanoparticle‐induced vascular blockade that increased tumor hypoxia (Zheng et al. 2024; Xu et al. 2024).

Prange et al. (2024) reported another approach for developing a DON prodrug by designing a redox‐responsive prodrug called redox‐DON. The structure of the redox‐DON molecule consists of a redox‐responsive disulfide linker at the N‐terminus that activates the drug in response to reducing conditions within the tumor microenvironment and tumor cells (Prange et al. 2024). Redox‐DON showed strong anti‐tumor efficacy in the CT26 mouse colon carcinoma model, with a significantly enhanced safety profile, particularly in the spleen and gastrointestinal tract (Prange et al. 2024).

Recently, a novel near‐infrared light‐activated DON‐TK‐BM3 prodrug was developed, which combines a cyanine dye (BM3) as a fluorophore linked to DON via a thioketal (TK) bond (Li et al. 2024). Upon radiation exposure, the photosensitive group BM3 generates ROS, which in turn breaks the TK linkage, allowing for an on‐demand release of DON (Li et al. 2024). The ROS‐induced cleavage in DON‐TK‐BM3 disrupts the intramolecular charge transfer, resulting in distinct fluorescence spectra between the coupled and uncoupled forms of BM3, enabling real‐time monitoring of prodrug activation (Li et al. 2024). DON‐TK‐BM3 demonstrated tumor‐specific activation and immune activation potential in A549 and H1975 non‐small cell lung cancer mouse xenograft models, with no toxicity toward normal cells (Li et al. 2024). Altogether, these studies demonstrate that the development of DON prodrugs is an active area of research that will hopefully enable efficient targeting of the HBP in clinical trials.

## 10 Future Directions to Accelerate Preclinical and Clinical Research Focused on the HBP

Drug development focused on targeting the HBP represents a promising and innovative strategy, with the potential to improve therapeutic options for tumors that are resistant to the current standard treatment. Prodrug development is being actively pursued to enhance the therapeutic properties and reduce the toxicity of DON (Lemberg et al. 2018), while nanocarriers have been explored to improve the solubility and cell permeability of the OGT inhibitors (Yang et al. 2022). Recognition of the therapeutic potential of targeting the HBP in cancer created a need for chemical and biochemical tools to study the effects of HBP inhibitors and the regulation of glycosylation and O‐GlcNAcylation. Substantial efforts are focused on developing assays that measure enzyme inhibition, kinetics, and binding affinity of the OGT inhibitors, as reviewed by Balsollier et al. (2021). The earliest method for assessing OGT activity utilized radiolabeled substrates. Currently, several commercially available luminescence‐ and fluorescence‐based assays, such as UDP‐Glo and PamStation, can be used to measure OGT activity and have been employed in high‐throughput screening studies to identify novel OGT inhibitors (reviewed by Balsollier et al. 2021; Alteen et al. 2021). Other methods that have been applied to measure the OGT activity in high‐throughput screening studies include fluorescence resonance energy transfer and homogeneous time‐resolved fluorescence assays (Gross et al. 2008; Wu et al. 2023). Recently, the latter has been applied for high‐throughput drug screening, which discovered CDDO (also called bardoxolone) as a novel OGT inhibitor with a distinct binding pocket from the previously reported OGT inhibitors (Wu et al. 2023). Several groups have also developed fluorescence polarization‐based assays to measure the binding affinity of OGT inhibitors (Gross et al. 2005; Rafie et al. 2018). Despite these advances, progress in identifying novel OGT inhibitors has been hindered by the lack of stability of the isolated OGT enzyme (Balsollier et al. 2021). As an alternative approach, a recent in silico study for drug repurposing identified a non‐steroidal anti‐inflammatory drug called celecoxib as a novel OGT inhibitor (Azevedo et al. 2024).

To further accelerate biological discovery on the role of the HBP, future efforts are needed to develop new assays for accurate monitoring of OGT and HBP enzyme activity in vitro, in vivo, and in tissue lysates. New advances are also needed to develop techniques for monitoring the cellular uptake of the HBP inhibitors (Balsollier et al. 2021; Alteen et al. 2021). There is also a demand for new chemical approaches to study the function of O‐GlcNAcylation and N‐/O‐linked glycosylation, and the effect of HBP inhibitors on these post‐translational modifications. In addition, current OGT inhibitors lack selectivity and disrupt the global O‐GlcNAc profile. Future research is expected to develop substrate‐specific OGT inhibitors (Estevez et al. 2020). These advances are expected to accelerate preclinical and clinical research focused on the HBP as a therapeutic vulnerability in cancer and sarcoma.

In this review, we provided evidence that overexpression of selected HBP enzymes is associated with patient outcomes; however, it remains unclear whether upregulation of the HBP enzymes also reflects increased sensitivity to pharmacological inhibition of this pathway. Expression of HBP enzymes is commonly elevated in cells that have adapted to metabolic or therapeutic stress. The HBP functions as a central hub that integrates these often concurrent stresses, thereby promoting tumor cell adaptation. It is not well understood whether the cells that managed to tolerate these conditions become dependent on the HBP, or whether increased expression of the HBP enzymes rather reflects cellular plasticity and reduced stress sensitivity. This question remains to be addressed in future functional studies.

## 11 Conclusions

In this review, we summarize the current state of knowledge on the role of HBP in sarcoma. The gene and protein expression levels of the HBP components have prognostic value in selected histological subtypes of sarcoma, suggesting that activation of this pathway may contribute to the aggressive behavior of these tumors. In sarcoma, fusion proteins may be predominantly modified by O‐GlcNAcylation, while oncoproteins may be modified mostly by N‐linked glycosylation. Glycosylation sites in the molecular drivers of sarcoma may occur mainly in the protein interaction, regulatory, and signaling domains. Based on the evidence of the anti‐tumorigenic effect of HBP inhibitors in preclinical cancer models, we propose that targeting the HBP in combination with chemotherapy, CDK inhibitors, or immunotherapy could potentially enhance therapeutic efficacy and help overcome resistance to these therapies in sarcoma. Finally, we highlight the need for further advances in basic biomedical research and drug development to better understand the role of the HBP and refine the pharmacological modulation of this pathway. The development of DON prodrugs is a rapidly advancing field of research that holds significant promise for enabling more precise and effective targeting of the HBP in clinical trials.

## Author Contributions

**Pegah Rahimizadeh** and **Richard Miallot:** conceptualization, data curation, formal analysis, investigation, visualization, writing – original draft, writing – review and editing, funding acquisition. **Chelsea De Bellis** and **Philippe Jolivet:** investigation, visualization, writing – review and editing. **Joanna Przybyl:** conceptualization, data curation, formal analysis, funding acquisition, investigation, methodology, project administration, supervision, writing – original draft, writing – review and editing.

## Conflicts of Interest

The authors declare no conflicts of interest.

## Supporting information


**Table S1:** DNA copy number alterations in the genes encoding the HBP components in the TCGA dataset of 206 specimens representing 6 histological subtypes of sarcoma.

**Table S2:** Protein domains of molecular drivers of sarcoma and benign mesenchymal tumors harboring glycosylation sites.

## Data Availability

Data sharing is not applicable to this article as no datasets were generated or analyzed during the current study.
