# Supplementary material for: Decoding the Hexosamine Biosynthesis Pathway: Implications for Novel Therapeutic Strategies in Sarcoma
Source: J Cell Physiol. 2026 May 11;241:e70182. doi: 10.1002/jcp.70182 (PMC13159415; doi:10.1002/jcp.70182)
Supplement: Supplementary file 1 — Table S1: DNA copy number alterations in the genes encoding the HBP components in the TCGA dataset of 206 specimens representing 6 histological subtypes of sarcoma. [file JCP-241-0-s002.docx]

**Table S1.** DNA copy number alterations in the genes encoding the HBP components in the TCGA dataset of 206 specimens representing 6 histological subtypes of sarcoma.

(Abbreviations: AMP – amplification; DEL – deletion; DDLPS - dedifferentiated liposarcoma; STLMS - soft tissue leiomyosarcoma; ULMS - uterine leiomyosarcoma; UPS - undifferentiated pleomorphic sarcoma; MFS - myxofibrosarcoma; SS - synovial sarcoma; MPNST - malignant peripheral nerve sheath tumors)

|  | **Total (n = 206)** | | **DDLPS (n = 50)** | | **STLMS (n = 53)** | | **ULMS (n = 27)** | | **UPS (n = 44)** | | **MFS (n = 17)** | | **SS (n = 10)** | | **MPNST (n = 5)** | |
| --- | --- | --- | --- | --- | --- | --- | --- | --- | --- | --- | --- | --- | --- | --- | --- | --- |
|  | AMP | DEL | AMP | DEL | AMP | DEL | AMP | DEL | AMP | DEL | AMP | DEL | AMP | DEL | AMP | DEL |
| ***GFPT1*** | 2 (1%) | - | - | - | - | - | 2 (7%) | - | - | - | - | - | - | - | - | - |
| ***GFPT2*** | 5 (2.5%) | 2 (1%) | 4 (8%) | - | 1 (2%) | - | - | - | - | 2 (5%) | - | - | - | - | - | - |
| ***GNPNAT1*** | 1 (0.5%) | 2 (1%) | - | 2 (4%) | - | - | 1 (4%) | - | - | - | - | - | - | - | - | - |
| ***PGM3*** | 1 (0.5%) | 1 (0.5%) | 1 (2%) | - | - | - | - | - | - | - |  | 1 (6%) | - | - | - | - |
| ***UAP1*** | 9 (4%) | 1 (0.5%) | 2 (4%) | 1 (2%) | - | - | 1 (4%) | - | 5 (11%) | - | 1 (6%) | - | - | - | - | - |
| ***GALE*** | 3 (1.5%) | 1 (0.5%) | - | - | - | - | - | - | 3 (7%) | - | - | 1 (6%) | - | - | - | - |
| ***OGT*** | 5 (2.5%) | - | 2 (4%) | 1 (2%) | 1 (2%) | 1 (2%) |  | 1 (4%) | 2 (5%) | 1 (2%) | - | - | - | - | - | - |
| ***OGA*** | 2 (1%) | - | - | - | - | - | - | 1 (4%) |  | - | 1 (6%) | - | - | - | - | - |
