# Supplementary material for: Decoding the Hexosamine Biosynthesis Pathway: Implications for Novel Therapeutic Strategies in Sarcoma
Source: J Cell Physiol. 2026 May 11;241:e70182. doi: 10.1002/jcp.70182 (PMC13159415; doi:10.1002/jcp.70182)
Supplement: Supplementary file 2 — Table S2: Protein domains of molecular drivers of sarcoma and benign mesenchymal tumors harboring glycosylation sites. [file JCP-241-0-s001.docx]

**Table S2.** Protein domains of molecular drivers of sarcoma and benign mesenchymal tumors harboring glycosylation sites.

(Abbreviations: FAD - flavin adenine dinucleotide)

| Protein name | Protein domains and regions harboring glycosylation sites |
| --- | --- |
| APC | Beta-catenin binding and downregulating domain, EB1 binding domain |
| ASPSCR1 | Low complexity region, UBX domain |
| β-catenin (CTNNB1) | Vinculin binding domain |
| COL1A1 | Fibrillar collagen C-terminal non-collagenous (NC1) domain |
| COL2A1 | Fibrillar collagen C-terminal domain |
| CREB1 | Kinase-inducible domain (KID) interacting domain, CREB-binding domain |
| DICER1 | Low complexity region |
| EWSR1 | Prion-like domain, Gly-rich domain, RNA binding domain |
| FGFR4 | Two immunoglobulin-like domains, protein kinase catalytic domain |
| FLI1 | Winged helix DNA-binding domain superfamily |
| FLT4 | Two immunoglobulin-like domains |
| FOXO1 | Transactivation domain |
| FUS | Low complexity region, RNA recognition domain, QCSY rich region, prion-like domain |
| HRAS | Small GTP-binding domain |
| JUN | JNK-mediated phosphorylation sites |
| KDR | Immunoglobulin-like domain |
| KIT | Immunoglobulin-like domain |
| LPP | Symbiosis region 1 |
| MTOR | PI3K/PI4K catalytic domain |
| MYC | N‑terminal transactivation domain myc homology box I |
| NCOA1 | Receptor-binding domain, nuclear receptor co-activator domain |
| NFIB | Transcription modulation region |
| PDGFB | PDGF/VEGF domain |
| POU5F1 | Lambda repressor like DNA-binding domain superfamily, homeobox domain, POU domain |
| PPFIBP1 | Pointed domain superfamily |
| PTPRB | PTP-type protein phosphatase domain |
| RAD51B | P-loop containing domain |
| RANBP2 | Zinc finger domain |
| SDHA | Catalytic domain, FAD binding domain |
